# ROS-GC interlocked Ca^2+^-sensor S100B protein signaling in cone photoreceptors: review

**DOI:** 10.3389/fnmol.2014.00021

**Published:** 2014-03-26

**Authors:** Rameshwar K. Sharma, Clint L. Makino, David Hicks, Teresa Duda

**Affiliations:** ^1^Research Divisions of Biochemistry and Molecular Biology, The Unit of Regulatory and Molecular Biology, Salus UniversityElkins Park, PA, USA; ^2^Department of Ophthalmology, Massachusetts Eye and Ear Infirmary, Harvard Medical SchoolBoston, MA, USA; ^3^Department of Neurobiology of Rhythms, Institute for Cellular and Integrative Neuroscience, CNRS UPR 3212Strasbourg, France

**Keywords:** ROS-GC guanylate cyclase, cyclic GMP, phototransduction, cones, S100B

## Abstract

Photoreceptor rod outer segment membrane guanylate cyclase (ROS-GC) is central to visual transduction; it generates cyclic GMP, the second messenger of the photon signal. Photoexcited rhodopsin initiates a biochemical cascade that leads to a drop in the intracellular level of cyclic GMP and closure of cyclic nucleotide gated ion channels. Recovery of the photoresponse requires resynthesis of cyclic GMP, typically by a pair of ROS-GCs, 1 and 2. In rods, ROS-GCs exist as complexes with guanylate cyclase activating proteins (GCAPs), which are Ca^2+^-sensing elements. There is a light-induced fall in intracellular Ca^2+^. As Ca^2+^ dissociates from GCAPs in the 20–200 nM range, ROS-GC activity rises to quicken the photoresponse recovery. GCAPs then progressively turn down ROS-GC activity as Ca^2+^ and cyclic GMP levels return to baseline. To date, GCAPs mediate the only known mechanism of ROS-GC regulation in the photoreceptors. However, in mammalian cone outer segments, cone synapses and ON bipolar cells, another Ca^2+^ sensor protein, S100B, complexes with ROS-GC1 and senses the Ca^2+^ signal with a K_1/2_ of 400 nM. Unlike GCAPs, S100B stimulates ROS-GC activity when Ca^2+^ is bound. Thus, the ROS-GC system in cones functions as a Ca^2+^ bimodal switch; with rising intracellular Ca^2+^, its activity is first turned down by GCAPs and then turned up by S100B. This presentation provides a historical perspective on the role of S100B in the photoreceptors, offers a pictorial model for the “bimodal” operation of the ROS-GC switch and projects future tasks that are needed to understand its operation. Some accounts of this review have been adopted from the original publications of these authors.

## INTRODUCTION

A seminal observation in the field of visual transduction was that a soluble bovine rod outer segment (ROS) fraction kicked the catalytic activity of a photoreceptor guanylate cyclase into high gear in the absence of Ca^2+^ (Koch and Stryer, 1988). The structure of this guanylate cyclase was believed to be composed of “separate regulatory and catalytic subunits” (Stryer, 1991); however, elucidating the molecular identity of the guanylate cyclase and its regulator turned out to be quite complicated. There were reports that guanylate cyclase possessed a molecular mass of 67 kDa and was nitric oxide sensitive (Horio and Murad, 1991a,b). Yet another report claimed to have solved the molecular structure of the photoreceptor retGC, the human retinal guanylate cyclase (Shyjan et al., 1992).

Later, the true photoreceptor ROS-guanylate cyclase (ROS-GC) was purified directly from bovine outer segments (Margulis et al., 1993) and its protein-sequence-based molecular cloning, structure, and function were established (Goraczniak et al., 1994). ROS-GC had a calculated molecular mass of 120,360 Da, a value similar to 112,000 Da reported earlier for a bovine (Koch, 1991) and 110,000–115,000 Da for toad photoreceptor guanylate cyclase (Hayashi and Yamazaki, 1991). Unlike the other known membrane guanylate cyclases, atrial natriuretic factor receptor guanylate cyclase (ANF-RGC; Paul et al., 1987; Sharma, 1988; Chinkers et al., 1989; Duda et al., 1991) and type C natriuretic peptide CNP-RGC guanylate cyclase (Koller et al., 1991; Duda et al., 1993), ROS-GC was not hormonally responsive (Goraczniak et al., 1994). It thus represented a new subfamily of the membrane guanylate cyclases. Further study revealed that ROS-GC was not composed of “separate regulatory and catalytic subunits”, it was not nitric oxide sensitive nor was its structure identical to retGC. The RetGC structure was eventually revised to match that of bovine ROS-GC (Accession number M92432).

Meanwhile, groups from the U.S. and the former Soviet Union joined forces to describe a newly purified Ca^2+^ binding protein, recoverin, that appeared to regulate guanylate cyclase activity (Dizhoor et al., 1991) and adhere to the functional description of Koch and Stryer (Koch and Stryer, 1988). It was therefore considered a Ca^2+^ modulator of ROS-GC; hence, named recoverin “because it promotes recovery of the dark state” (Dizhoor et al., 1991). However, ever purer preparations of recoverin showed a disturbing decline in guanylate cyclase stimulation. It became evident that recoverin was not the sought after regulator of guanylate cyclase activity and the initial conclusion was withdrawn (Hurley et al., 1993). Instead, recoverin exerted Ca^2+^-dependent control over the phosphorylation of photoexcited rhodopsin by rhodopsin kinase (Chen et al., 1995; Klenchin et al., 1995; Kawamura, 1999). At almost the same time, two separate groups discovered similar but distinct guanylate cyclase activating proteins (GCAPs), GCAP1 (Palczewski et al., 1994; Subbaraya et al., 1994; Gorczyca et al., 1995; Frins et al., 1996) and GCAP2 (Dizhoor et al., 1995). These GCAPs did indeed stimulate ROS-GC activity as described by Koch and Stryer (1988).

Recombinant ROS-GC expressed in a heterologous system of COS cells responded at 10 nM [Ca^2+^]_i_ to GCAP1 stimulation in a dose-dependent manner (Duda et al., 1996b). The stimulation was inhibited cooperatively by free Ca^2+^ with a K_1/2_ of 100 nM. Under identical conditions, GCAP1 had no effect on recombinant ANF-RGC, the peptide hormone receptor guanylate cyclase. An important characteristic of the transduction system was that GCAP remained bound to ROS-GC at low and high Ca^2+^ levels, consistent with physiological observations (Koutalos et al., 1995). Similar reconstitution studies established GCAP2 as another Ca^2+^-sensing subunit of ROS-GC (Dizhoor et al., 1995; Gorczyca et al., 1995). When a second ROS-GC was subsequently discovered in the bovine retina, termed Ret-GC2 in human retina (Lowe et al., 1995), it was named ROS-GC2 to distinguish it from the original ROS-GC which was renamed as ROS-GC1 (Goraczniak et al., 1997). It became clear that a Ca^2+^-modulated system, composed of a pair of ROS-GCs and a pair of GCAPs, subserves phototransduction in the outer segments of photoreceptors. This conclusion was supported at the physiological level by the studies with the double knockout ROS-GC1/ROS-GC2 mouse model (Baehr et al., 2007). Rods and cones were non-functional, excluding the presence of a third guanylate cyclase linked with phototransduction.

Thus ROS-GCs are related to peptide hormone receptor guanylate cyclases but they do not respond to extracellular ligands. Instead, they exist in stable complexes with GCAPs that sense changes in internal [Ca^2+^]. The feature that incrementing [Ca^2+^]_i_ progressively reduces ROS-GC1 catalytic activity was academically challenging because such a mechanism had never been observed before for any of the members of the guanylate cyclase family. All other members were solely stimulated by their effector ligands, natriuretic peptide hormones, at their extracellular domains. The molecular properties of this remarkable system and the role that the system plays in phototransduction are described in greater depth in Pugh et al. (1997), Sharma and Duda (2012).

## DISCOVERY OF CD-GCAP MEANT THAT ROS-GC1 COULD OPERATE AS A Ca^**2+**^ BIMODAL TRANSDUCTION SWITCH

Contemporaneous with the discovery of GCAPs that stimulated ROS-GC1 at low Ca^2+^, a post-mitochondrial 100,000 *g* supernatant fraction from retina was found to stimulate recombinant ROS-GC at high Ca^2+^ by as much as 25-fold (Pozdnyakov et al., 1995). The new factor was a peptide with an apparent molecular weight of 6–7 kDa protein that oligomerized to a functional size of 40 kDa. It stimulated native and recombinant ROS-GC with a K_1/2_ for Ca^2+^ of 2 μM with biochemical characteristics typical of a Ca^2+^-binding protein. To distinguish it from the just-discovered GCAPs, the new factor was named CD-GCAP (Ca^2+^-dependent guanylate cyclase activator protein; Pozdnyakov et al., 1995).

**FIGURE 1:**
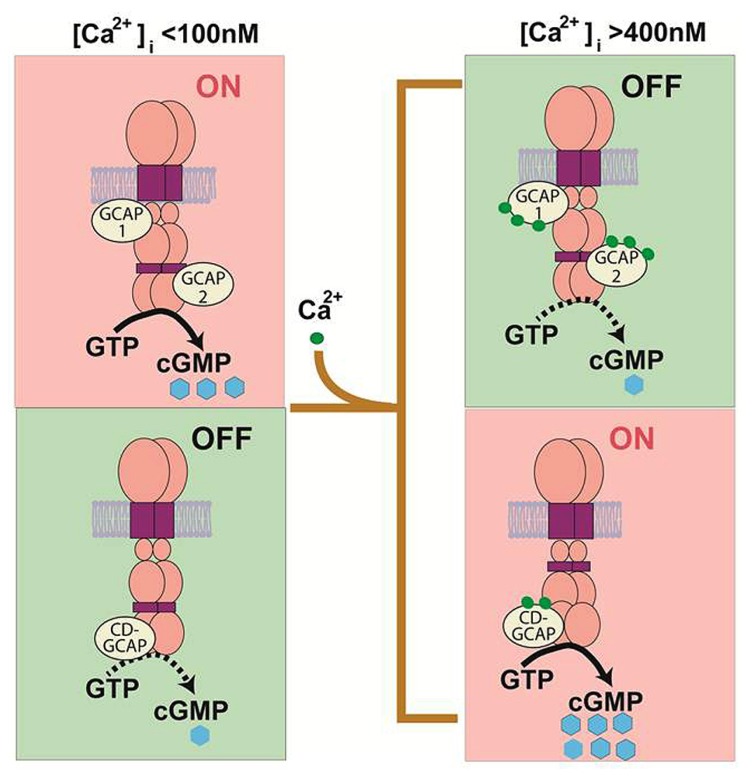
**Original theoretical Ca^**2+**^ bimodal ROS-GC transduction model.** In the absence of bound Ca^2+^, GCAPs 1 and 2 boost ROS-GC1 catalytic activity. In contrast, S100B boosts ROS-GC1 activity in the Ca^2+^-bound state. GCAPs sense [Ca^2+^]_i_ < 200 nM while S100B senses [Ca^2+^]_i_ > 200 nM. Thus in the GCAPs operational mode the S100B switch is “OFF” and in the S100B operational mode the GCAP switch is “OFF” (Upgraded with permission from Sharma, 2010; Sharma and Duda, 2012).

The capacity for ROS-GC to couple to GCAPs or to CD-GCAP allows for a cell to sculpt its response to Ca^2+^. Depending upon the cell’s specific needs, it could express either GCAP or CD-GCAP to stimulate cyclic GMP synthesis at low (tens of nanomolar) or at high (sub-micromolar) ranges of [Ca^2+^]. An even more exciting possibility exists for the simultaneous expression of both Ca^2+^-regulatory subunits in the same cell. ROS-GC could then operate as a Ca^2+^-bimodal transduction switch, stimulated at low and at high free [Ca^2+^] by Ca^2+^ -free GCAPs and by Ca^2+^-bound S100B, respectively. A theoretical Ca^2+^-BIMODAL ROS-GC transduction model embodying these features was proposed (**Figure 1**). Bimodal Ca^2+^ sensing could be an elegant general mechanism for neural transmission.

## CD-GCAP IS AN S100 PROTEIN

The first indication that CD-GCAP might be related to S100 proteins was that it appeared as a 6–7 kDA Ca^2+^-binding protein on SDS-PAGE (Pozdnyakov et al., 1995). S100 proteins are known to run anomalously as 6–7 kDa proteins in SDS-PAGE even though their subunit molecular weight, calculated from amino acid (aa) sequence, is about 10.5 kDa (Kligman and Marshak, 1985). Additional evidence for the structural similarity of CD-GCAP to S100 emerged from mass spectrometry analysis (Pozdnyakov et al., 1997). The fragmented molecular masses of 10,580 Da for CD-GCAP and 10,582 Da for S100B were essentially the same. Tryptic digests also yielded indistinguishable fragmentation patterns. Finally, bovine CD-GCAP and bovine S100B were cloned and their predicted primary protein structures were identical (Pozdnyakov et al., 1997).

Despite being structurally identical proteins, biochemical analyses revealed two surprising functional discrepancies. As expected, S100A1–S100B and S100ββ dimer (S100B) from commercial sources stimulated cloned recombinant and wt-photoreceptor ROS-GC (Duda et al., 1996a; Margulis et al., 1996). But the V_max_ of ROS-GC was about 50% higher when activated by CD-GCAP than when activated by commercially obtained S100B. In addition, the commercial S100B required about 20-times more calcium (3.2 x 10^-5^ M vs. 1.5 x 10^-6^ M for CD-GCAP) for half-maximal stimulation of guanylate cyclase. Clearly, CD-GCAP was not quite the same as the commercially obtained S100B.

In the search for the explanation for these functional differences, the proteins were analyzed for post-translational modifications and higher order structures (Pozdnyakov et al., 1997). After treating both proteins with hydroxylamine, a deacylating agent, CD-GCAP no longer activated guanylate cyclase while S100B activation retained its ability to do so. Furthermore, hydroxylamine fragmented CD-GCAP while S100B was unaffected. These results indicated that the conformations of the two proteins were different. Such a difference could have arisen from variations in the procedures for purifying the two proteins: CD-GCAP purification entailed heating at 75°C in 5 mM Ca^2+^, while S100B purification included zinc affinity chromatography (commercial sources refused to provide these details but their references indicated so). To investigate, CD-GCAP was subjected to zinc affinity chromatography (CD-GCAP-to-S100B) and S100B was heated to 75°C in Ca^2+^ (S100B-to-CD-GCAP). Guanylate cyclase activation, calcium–sensitivity, and hydroxylamine lability measurements demonstrated that CD-GCAP-to-S100B was identical to S100B and that S100B-to-CD-GCAP was identical to CD-GCAP. Apparently zinc inhibits the native S100B potency toward stimulation of ROS-GC. An additional finding was that S100B monomer is about twice as effective as the dimer in activating the ROS-GC catalytic activity. Hence, CD-GCAP and commercially obtained S100B are conformational isomers. The native form of S100B present in the retinal neurons is CD-GCAP.

## CHARACTERIZATION OF THE S100B BINDING SITE OF ROS-GC1

Direct interaction between ROS-GC1 and S100B was verified by cross-linking experiments with bis-(sulfosuccinimidyl)suberate. A cross-linked complex, consisting of ROS-GC1 dimer and S100B, was detected by both ROS-GC1 and S100B antibodies on Western blots (Duda et al., 2002). Biochemical experiments with ROS-GC1 deletion constructs localized an interaction of S100B with the C-terminus of ROS-GC1, aa 731–1054 (Duda et al., 1996a). An EC_50_ of 395 nM, measured using surface plasmon spectroscopy, was comparable to the EC_50_ of 800 nM for stimulation of ROS-GC1 by S100B in a biochemical assay (Duda et al., 2002).

Since the aa 733–964 segment containing the putative dimerization and catalytic domains is highly conserved, the less conserved aa 965–1054 segment was subjected to further analyses. First, three ROS-GC1 deletion mutants: Δ965–1054, Δ972–1054, and Δ1016–1054 were analyzed for their responses to S100B. The first mutant was totally unresponsive, the response of the second mutant was only ~25% and that of the third mutant ~50% of the wild-type ROS-GC1 response to S100B. Therefore the entire region aa 965–1054 of ROS-GC1 is important for Ca^2+^-dependent stimulation by S100B, but the region aa 965–1016 appears to be the most critical (Duda et al., 2002). Second, overlapping peptides encompassing this region were screened for their effects on S100B-dependent stimulation of ROS-GC1 at high Ca^2+^. Two peptides covering aa regions 952–991 and 1029–1040 prevented ROS-GC1 stimulation by S100B. Finer analysis revealed that aa 962–981 formed the critical binding site of ROS-GC1 with half-maximal binding at 198 nM. A ^966^RIHVNRS^972^ motif was obligatory, however, a flanking cluster of four aa residues, R^1039^RQK^1042^ formed a low affinity binding site. While not absolutely required for binding, the secondary site was an S100B-modulated transduction site that conferred a twofold increase in maximal activation of ROS-GC1 (Duda et al., 2002). 

These studies validated the unique ability of ROS-GC1 to respond not only to low Ca^2+^ signals mediated by GCAPs but also to high Ca^2+^ signals mediated by S100B. To analyze the possible interplay of these signals, the aforementioned ROS-GC1 deletion mutants were analyzed for their responses to GCAP1 (Duda et al., 2002) and GCAP2 (Duda et al., 2005). At 10 nM Ca^2+^, activation of all mutants was identical to that of wild-type ROS-GC1. Thus, the S100B and GCAP1 regulatory sites of ROS-GC1 are distinct and do not interfere with one other. This result was not surprising because the binding site for GCAP1 was previously mapped to the N-terminal part of the ROS-GC1 cytoplasmic domain (Duda et al., 1999; Lange et al., 1999).

**FIGURE 2:**
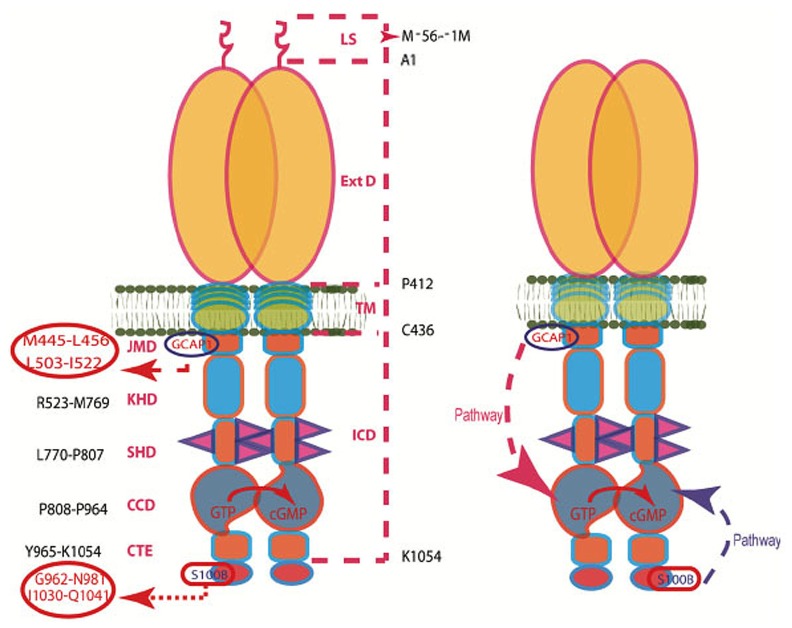
**The modular nature of ROS-GC1.** *Left Panel*. The dashed lines on the right show the domain boundaries: LS, leader sequence (note: the leader sequence is absent from the mature protein shown in the right panel); ExtD, extracellular domain; TM, transmembrane domain; ICD, intracellular domain. The designated names and the amino acid residues constituting their boundaries are indicated on the left: JMD, juxtamembrane domain housing the indicated GCAP1-binding site; KHD, kinase homology domain; SHD, signaling helix domain; CCD, core catalytic domain; CTE, C-terminal extension housing the S100B-binding site. It is noteworthy that the ICD contains all of the functional domains and that GCAP1 and S100B target sites on opposite sides of the CCD*. Right panel*. The GCAP1 and S100B signaling pathways within ROS-GC propagate in opposite directions. The trajectory of the GCAP1 pathway is shown with the red dashed arrow. From its origin in the JMD, it passes “downstream” through the structural KHD and SHD in its course to the CCD. The trajectory of the S100B pathway shown with the blue dashed arrow. Originating in the CTE, it directly flows “upstream” to the CCD. The CCD exists as an antiparallel homodimer. Both GCAP1 and S100B signals are translated at the CCD into faster production of cyclic GMP (Modified from Duda et al., 2012).

The case for GCAP2 was not so clear. Initial mapping had localized the GCAP2 binding site closer to the C-terminus of ROS-GC1 (Krishnan et al., 1998). In reconstitution experiments with the ROS-GC1 deletion mutants (the same as used for mapping the S100B site: *vide supra*), the Δ965–1054 mutant failed to respond to GCAP2, the Δ972–1045 responded with V_max_ of ~50% of that of the wild-type ROS-GC1, and the response of the Δ1016–1054 mutant matched that of the wild-type cyclase, indicating that the functional GCAP2-modulated domain resided within aa 965–1016. Detailed peptide competition assays narrowed down the GCAP2-modulated site to the sequence motif Y^965^-N^981^ (Duda et al., 2005). As expected, this site is distinct from the GCAP1 site. It, however, partially overlaps with the S100B-regulatory site. These results provide a hint that, when GCAP1 and S100B are co-expressed, a single ROS-GC1 complex could operate as a bimodal Ca^2+^ switch. On the other hand, when GCAP2 and S100B are co-expressed, the switch might involve separate populations of ROS-GC1 complexes. Furthermore, identification of the binding sites for the Ca^2+^ sensors disclosed an intriguing topographical feature of ROS-GC1; GCAP2 and S100B transmit the Ca^2+^ signals to the catalytic domain of ROS-GC1 from the C-terminal side while GCAP1 does so from a more distant location on the N-terminal side. These intramolecular mechanics are specific to ROS-GC1 and are different from the peptide hormone receptor subfamily, where the signal to the catalytic site migrates solely downstream from a site on the N-terminus located on the opposite side of the membrane. A schematic model of the system is depicted in **Figure 2**.

ROS-GC2 has an ~8x lower affinity for S100B and with S100B bound at high Ca^2+^, its maximal activation is twofold lower than that of ROS-GC1. Substitution of aa 731–1053 of ROS-GC1 into the corresponding region of ROS-GC2 and vice versa reversed the selectivity (Duda et al., 1998). These results suggest that ROS-GC2 may be an S100B partner of lesser importance than ROS-GC1.

## S100B IS EXPRESSED IN CONES BUT NOT IN RODS

Guided by the knowledge that ROS-GC and GCAPs are present in photoreceptors, including their outer segments where they serve as critical components of phototransduction (Cuenca et al., 1998; Kachi et al., 1999; reviewed in Pugh et al., 1997; Koch et al., 2010; Sharma and Duda, 2012), and that S100B is also present in photoreceptors (Rambotti et al., 1999; reviewed in: Rambotti et al., 2002), questions were raised as to whether a ROS-GC1 bimodal Ca^2+^ -regulated system operates in visual transduction and whether it is present in both rods and cones. These issues are being addressed in mouse (e.g., Wen et al., 2012), for which there exist useful knockouts including S100B^-/-^, GCAP1^-/-^, GCAP2^-/-^, GCAP1/GCAP2^-/-^, ROS-GC1^-/-^.

Affinity purified S100B antibody recognized on Western blots of mouse photoreceptors, a protein of molecular weight corresponding to S100B. In biochemical assays, guanylate cyclase activity of mouse ROS membranes exhibited bimodal Ca^2+^ switch behavior; activity decreased as [Ca^2+^] rose from 10 to 300 nM and then increased as [Ca^2+^] began to exceed 300 nM. The “inhibitory” phase of the guanylate cyclase activity was consistent with the loss of ROS-GC1 activation by GCAPs upon Ca^2+^ binding to the latter and the stimulatory phase with Ca^2+^-induced activation of ROS-GC1 activity by S100B. The increase in ROS-GC activity at low [Ca^2+^] disappeared in ROS membranes from GCAP1/GCAP2^-/-^ mice, whereas the increase in ROS-GC activity at high [Ca^2+^] was missing in ROS membranes from S100B^-/-^ mice and from ROS-GC1^-/-^ mice. The significance of the result from ROS-GC1^-/-^ mice will be revealed below. Yet, regulation of ROS-GC1 by S100B was not functionally linked to phototransduction in rods because flash responses from wild-type and S100B-KO rods were indistinguishable (Wen et al., 2012). The possibility that S100B might contribute to the rod photoresponse at high free [Ca^2+^] outside the normal physiological range was tested and not found.

**FIGURE 3:**
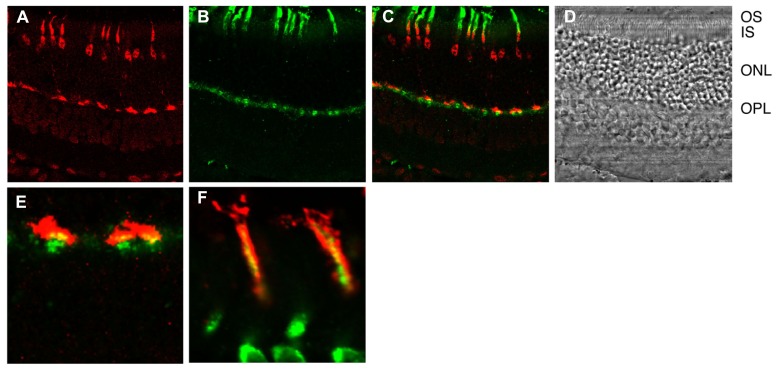
**Immunhistochemical localization of S100B to cones in the murine retina.** **(A)** Cone arrestin in red. Cone outer segment labeling is overshadowed with by strong labeling of the synapse, nucleus and inner segment. **(B)** S100B in green. Selected outer segments are labeled intensely. Inner segments and synapses are also labeled. **(C)** Composite showing that cone arrestin and S100B labeling are restricted to cones. **(D)** Bright field. OS, outer segments; IS, inner segments; ONL, outer nuclear layer containing nuclei of rods and cones; OPL, outer plexiform layer containing synapses of rods and cones. **(E)** Composite showing cone synapses at higher magnification. Overlap (yellow) between arrestin and S100B is not perfect because the former is a soluble protein whereas the latter is membrane bound. **(F)** Section from a different retina that was doubly labeled for cone arrestin in green and S100B in red at high magnification. Conditions were adjusted to optimize the detection of outer segment labeling Courtesy of A. Pertzev, Salus University.

Detailed histochemical analysis of S100B expression in the mouse retina brought all these observations into accord (**Figure 3**). As expected, the S100B-KO retina showed no S100B immunoreactivity (Wen et al., 2012), affirming the specificity of the S100B antibody, because photoreceptor outer segments also express S100A1, another member of the S100 family (Rambotti et al., 1999). S100B immunoreactivity was present in wild-type photoreceptors, however, it was not present in all of them. The scattered S100B staining corresponded to that for cone-specific arrestin. On the other hand, in retinas of Nrl^-/-^ mice, all outer segments labeled for S100B as well as for cone arrestin. Because Nrl^-/-^ retina is rod-less and populated exclusively with cone photoreceptors (Mears et al., 2001) these results strongly indicate that among photoreceptors, S100B expression is restricted to cones. Unimodal switch behavior in ROS-GC1^-/-^ outer segments (above) is now easy to understand. In ROS-GC1^-/-^ retinas, cones degenerate but the rods remain intact (Yang et al., 1999). Rods however, do not express S100B, so the only Ca^2+^-regulated guanylate cyclase activity in ROS-GC1^-/-^ retinas is the GCAPs modulated ROS-GC2 activity.

Our ongoing immunohistochemical studies with the Nile Rat (*Arvicanthis ansorgei*) retina in which 33% of the photoreceptors are cones, in contrast to the 3% cone population in the mouse (Bobu et al., 2006) appear to corroborate the conclusion derived from analyses of murine retinas that S100B co-exists with ROS-GC1 exclusively in the cone outer segments.

**FIGURE 4:**
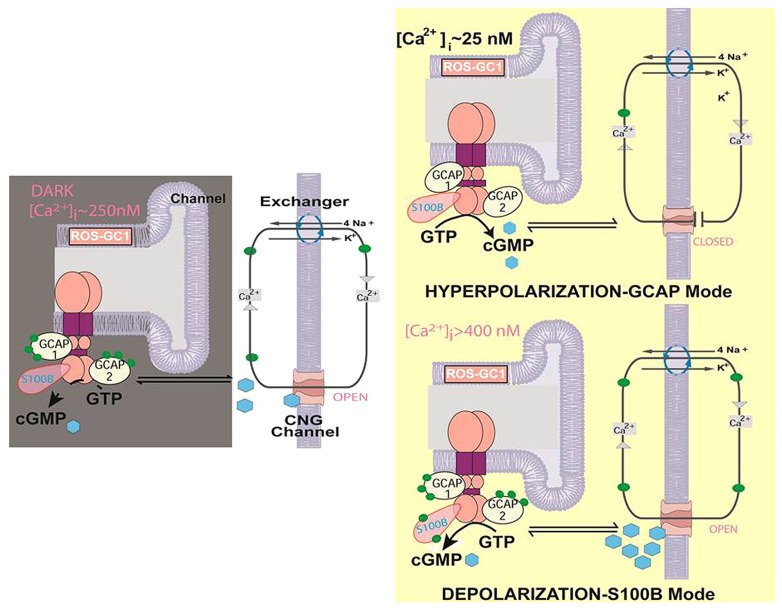
**Two modes of ROS-GC1 modulation by [Ca^**2+**^]_**i**_ in cones.** *Dark state [left]*. At [Ca^2+^]_i_ ~250 nM Ca^2+^ sensors: GCAP1, GCAP2, S100B are ROS-GC1 bound; GCAP1 and GCAP2 are [Ca^2+^]_i_ bound; ROS-GC1 is in the basal state. Cyclic GMP generated keeps a fraction of CNG channels open allowing an influx of Na^+^ and Ca^2+^. In this graphical simplification, GCAP2 and S100B are shown bound to the same ROS-GC dimer but it is more likely that these two Ca^2+^ sensors bind separate dimers. *Hyperpolarization-GCAP mode [Top right].* LIGHT triggers activation of phosphodiesterase and hydrolysis of cyclic GMP. The decrease in cyclic GMP causes CNG channels to close, preventing influx of Na^+^ and Ca^2+^ and hyperpolarizing the outer segment plasma membrane. Extrusion of Ca^2+^ by the Na^+^/Ca^2+^, K^+^ exchanger lowers [Ca^2+^]_i_ from ~250 nM to about 25 nM. The decline causes GCAPs to stimulate ROS-GC1. *Depolarization-S100B mode [Bottom right]*. [Ca^2+^]_i_ levels rise when CNG channels open. With K_1/2_ of 400 nM, S100B captures Ca^2+^, triggering activation of ROS-GC1. Cyclic GMP formed could open many more CNG channels, greatly increasing the influx of Na^+^ and Ca^2+^. [Ca^2+^]_i_ levels rise still further because extrusion of Ca^2+^ through Na^+^/Ca^2+^, K^+^ exchanger slows as the ion gradients collapse. The cone outer segment may lose the ability to maintain a membrane potential (Modified from Wen et al., 2012).

At present, the question about the role of the ROS-GC1 bimodal switch in cone phototransduction is still unanswered because cones from S100B^-/-^ mice have yet to be recorded. A model illustrating some of the difficulties in understanding the operation of the bimodal switch is shown in **Figure 4**. One issue is that in darkness (DARK), Ca^2+^ will not bind to S100B and activate the switch if [Ca^2+^]_i_ never exceeds 250 nM. However, the value of 250 nM for [Ca^2+^]_i_ in darkness was taken from mouse rods (Woodruff et al., 2002) because measurements from mouse cones have not yet been made. Thus the true [Ca^2+^]_i_ for cones in darkness may be higher. A second issue is that without proper restraint, operation of the bimodal switch at high [Ca^2+^]_i_ would ignite an explosive positive feedback: increased cyclic GMP production, followed by opening of more cyclic GMP-gated channels, which then allow a greater influx of Ca^2+^, etc. Cones could accumulate lethal levels of Ca^2+^ or experience a collapse in ion gradients (DEPOLARIZATION-S100B Mode). Indeed, genetic mutations that result in a hyperactive ROS-GC complex cause the photoreceptors to degenerate (Olshevskaya et al., 2002; Zägel and Koch, 2014).

## THE BIMODAL SWITCH EXISTS AT THE PHOTORECEPTOR-BIPOLAR CELL SYNAPSE

Immunohistochemical studies also place ROS-GC1, GCAP1 and S100B in the synaptic layers of the retina (Liu et al., 1994; Cooper et al., 1995; Duda et al., 2002; Venkataraman et al., 2003). To test whether the ROS-GC1 transduction system utilizes a Ca^2+^ bimodal switch at the photoreceptor-bipolar cell synapse (presumably the cone to bipolar synapse, see **Figure 3**), bovine retinal synaptosomes (P1) were prepared and analyzed. Purity of the preparation was ascertained by Western blotting with rhodopsin kinase antibody. No immunoreactivity for rhodopsin kinase was detected, assuring that the preparation was not contaminated with ROSs, the original residential site of the ROS-GC1. Western blots were positive for ROS-GC1, GCAP1 and S100B demonstrating that indeed the synaptosomal fraction contained all the components of the ROS-GC1 bimodal system and was suitable for functional analyses.

To be physiologically relevant, the components of the ROS-GC1 bimodal system, ROS-GC1, GCAP1 and S100B must reside in proximity and not, e.g., in different populations of synaptosomes. Because the Western analyses described above did not provide information about the proximity of ROS-GC1, GCAP1 and S100B molecules, immunohistochemical analyses (Duda et al., 2002; Venkataraman et al., 2003) and cross-linking experiments were performed.

Cryosections of bovine retina were doubly labeled with antibodies against GCAP1 or ROS-GC1 and SV2, a synaptic vesicle protein that strongly stains photoreceptor termini (Buckley and Kelly, 1985). The results revealed co-expression of ROS-GC1 and GCAP1 in cone synaptic termini (Venkataraman et al., 2003). Similarly, co-localization of ROS-GC1 and S100B was shown (Duda et al., 2002).

Cross-linking experiments with a non-cleavable imidoester confirmed the proximity of ROS-GC1 and GCAP1 in the synaptosomal fraction (Venkataraman et al., 2003). Without the cross-linker, the ROS-GC1 and GCAP1 antibodies identified separate bands at ~120 kDa and ~21 kDa, respectively, corresponding to the appropriate monomers. But in presence of the cross-linker, both GCAP1 and ROS-GC1 antibodies identified a common immunoreactive band at ~250 kDa, the approximate size for a ROS-GC1 dimer-GCAP1 complex. The results demonstrated that ROS-GC1 and GCAP1, indeed, resided in proximity. Because ROS-GC1 resides with GCAP1 in cone synaptic terminals and ROS-GC1 also resides with S100B, it was inferred that all three components of the bimodal system co-reside in the photoreceptor synaptic terminals, specifically cone synaptic terminals.

Functionality of the ROS-GC1 bimodal switch in the P1 fraction was determined by measuring guanylate cyclase activity at Ca^2+^ concentrations ranging from 10 nM to 10 μM. Below 200 nM, Ca^2+^ elicited a dose-dependent decrease in guanylate cyclase activity with an “inhibitory” value (IC_50_) for Ca^2+^ of 100 nM. But at higher concentrations, ROS-GC activity began to climb with an EC_50_ of 0.8 μM. Side by side analysis of photoreceptor outer segment and P1 membranes demonstrated that the cyclic GMP synthetic activities were inhibited identically by free Ca^2+^ with an IC_50_ of 100 nM.

That GCAP1 was involved in the “inhibitory” phase of ROS-GC1 activity was confirmed by peptide competition experiments. A sequence motif L^503^–I^522^ in ROS-GC1 is critical and specific for the cyclase activation by GCAP1 (Lange et al., 1999). The core motif within this domain consists of D^507^–R^518^. Analysis of the P1 and photoreceptor outer segments membranes in parallel demonstrated that the incremental concentrations of the L^503^–I^522^ and D^507^–R^518^ peptides at 10 nM Ca^2+^ inhibited their guanylate cyclase activities with the same dose-dependence. A control peptide of L^503^–I^522^ with a scrambled sequence had no effect on GCAP-dependent ROS–GC1 activity. Both fractions showed a reversal of the peptide (D^507^–R^518^)-dependent inhibition upon the addition of an excess of GCAP1 (Venkataraman et al., 2003). Thus Ca^2+^ signaling in the synapse region is modulated by GCAP1 and S100B. GCAP1 has the higher affinity for Ca^2+^ and upon binding, lowers ROS-GC1 activity. S100B captures the high Ca^2+^ signal and upon binding, stimulates ROS-GC1.

## A ROLE IN SIGNAL PROCESSING IN THE RETINA?

ERG recordings from wild-type and S100B^-/-^ mice provided the first clues as to the physiological significance of S100B in retinal function (Wen et al., 2012). The ERG is a field potential across the retina generated by the electrical activity of all cells, including that of rods and cones (reviewed in: Peachey and Ball, 2003; Weymouth and Vingrys, 2008). The corneal negative a-wave, which sums the photocurrent responses of rods, is normal in the S100B-KO mice because S100B is not present in rods. The corneal positive b-wave, generated mainly by ON-bipolar cells, displays slower kinetics in S100B^-/-^ mice; it peaks later and takes longer to recover. Oscillatory potentials that arise from the activity of inner retinal neurons are smaller and there are disturbances in their timing. Gaps in our knowledge about what S100B is doing in the cone outer segment, the cone synapses and for that matter, in other retinal neurons preclude any attempt to interpret the changes in the ERG at this time.

## FUTURE DIRECTIONS

The composition and the operational principles of cones and rods differ in ways that support their specialized visual functions: extremely high sensitivity in rods, rapid responsiveness over a wide dynamic range in cones. In addition to the GCAPs, S100B serves as an additional Ca^2+^ sensor component of ROS-GC1 in cones. Future tasks will be to decode this signal transduction system at the molecular and physiological levels, to link it with cone-related dystrophies and to design therapeutic options. Progress would be facilitated if several important objectives were to be met. (1) A suitable mammalian model with abundant cones needs to be selected. No such mouse model is available at present. Through genetic manipulation, development of the rods has been suppressed in Nrl^-/-^ mice and their retinas are cone-exclusive. But these cones may not be exact copies of the natural, although biochemically and physiologically, they appear to be very close (Nikonov et al., 2005, 2006). One alternative is the Nile rat (*A. ansorgei*), whose retina contains about 30% cones in contrast to the 3% population in the mouse (Bobu et al., 2006). (2) The molecular architecture of the ROS-GC complex needs to be understood. Which combinations of Ca^2+^ sensor proteins are possible in the complex? Which actually occur? How are their relative levels within different cell types regulated? (3) Ca^2+^-modulated signal transduction by the ROS-GC complex needs to be defined at a molecular level. For the S100B native to cones, what are the kinetic parameters of the S100B transduction switch? By what mechanism does S100B sense Ca^2+^ and how does it effect a change in the catalytic domain of ROS-GC? (4) The physiological role of S100B in the cone photoresponse needs to be assessed. Does it accelerate the recovery of cone response and increase the dark current? What limits S100B stimulated ROS-GC activity at high Ca^2+^? Does S100B increase the rate of vesicular release of neurotransmitter at the synapse? S100A1 is also expressed in outer segments, what is its function? (5) S100B is expressed in inner retinal neurons but nothing is known about what it does there. (6) S100B protein does not fit in the presently classified NCS protein family, sharing only 15% sequence identity and containing only two EF-hands. Therefore, the NCS family needs to be expanded to include S100B as a branch.

## Conflict of Interest Statement

The authors declare that the research was conducted in the absence of any commercial or financial relationships that could be construed as a potential conflict of interest.
